# Cross-Cultural Sense-Making of Global Health Crises: A Text Mining Study of Public Opinions on Social Media Related to the COVID-19 Pandemic in Developed and Developing Economies

**DOI:** 10.2196/58656

**Published:** 2025-01-27

**Authors:** Adham Kahlawi, Firas Masri, Wasim Ahmed, Josep Vidal-Alaball

**Affiliations:** 1 Department of Statistics, Computer Science, Applications University of Florence Florence Italy; 2 Lloyds Banking Group (United Kingdom) Edinburgh United Kingdom; 3 Newcastle Business School Northumbria University Newcastle Upon Tyne United Kingdom; 4 Hull Univeristy Business School Hull University Kingston Upon Hull United Kingdom; 5 Unitat de Recerca i Innovació Gerència d'Atenció Primària i a la Comunitat de la Catalunya Central Institut Català de la Salut Sant Fruitós de Bages Spain; 6 Intelligence for Primary Care Research Group Unitat de Suport a la Recerca de la Catalunya Centra Fundació Institut Universitari per a la recerca Sant Fruitós de Bages Spain; 7 Department of Medicine Faculty of Medicine University of Vic Vic Spain

**Keywords:** COVID-19, SARS-CoV-2, pandemic, citizen opinion, text mining, LDA, health crisis, developing economies, Italy, Egypt, UK, dataset, content analysis, social media, twitter, tweet, sentiment, attitude, perception, perspective, machine learning, latent Dirichlet allocation, vaccine, vaccination, public health, infectious

## Abstract

**Background:**

The COVID-19 pandemic reshaped social dynamics, fostering reliance on social media for information, connection, and collective sense-making. Understanding how citizens navigate a global health crisis in varying cultural and economic contexts is crucial for effective crisis communication.

**Objective:**

This study examines the evolution of citizen collective sense-making during the COVID-19 pandemic by analyzing social media discourse across Italy, the United Kingdom, and Egypt, representing diverse economic and cultural contexts.

**Methods:**

A total of 755,215 social media posts from X (formerly Twitter) were collected across 3 time periods: the virus' emergence (February 15 to March 31, 2020), strict lockdown (April 1 to May 30, 2020), and the vaccine rollout (December 1, 2020 to January 15, 2021). In total, 284,512 posts from Italy, 261,978 posts from the United Kingdom, and 209,725 posts from Egypt were analyzed using the latent Dirichlet allocation algorithm to identify key thematic topics and track shifts in discourse across time and regions.

**Results:**

The analysis revealed significant regional and temporal differences in collective sense-making during the pandemic. In Italy and the United Kingdom, public discourse prominently addressed pragmatic health care measures and government interventions, reflecting higher institutional trust. By contrast, discussions in Egypt were more focused on religious and political themes, highlighting skepticism toward governmental capacity and reliance on alternative frameworks for understanding the crisis. Over time, all 3 countries displayed a shift in discourse toward vaccine-related topics during the later phase of the pandemic, highlighting its global significance. Misinformation emerged as a recurrent theme across regions, demonstrating the need for proactive measures to ensure accurate information dissemination. These findings emphasize the role of cultural, economic, and institutional factors in shaping public responses during health crises.

**Conclusions:**

Crisis communication is influenced by cultural, economic, and institutional contexts, as evidenced by regional variations in citizen engagement. Transparent and culturally adaptive communication strategies are essential to combat misinformation and build public trust. This study highlights the importance of tailoring crisis responses to local contexts to improve compliance and collective resilience.

## Introduction

### Background

According to the World Health Organization, COVID-19 was one of the most significant global public health challenges. Italy, Egypt, and the United Kingdom were among the countries considered to be the worst affected [1,2]. Many studies have noted how the internet and social media are essential in communicating risks and emergencies before, during, and after a crisis [3,4]. Human behavior related to using and processing information has been enormously affected by the orientations of the digital world in the 21st century. This uniquely contrasts previous global deadly disease outbreaks such as the Black Death and the Spanish Influenza. Furthermore, a recent study by Christianson & Barton noted how COVID-19 had created a unique scenario that provides an opportunity to study “sense-making” [4].

Digital platforms allow users to converse with one another for collective sense-making, which involves seeking, generating, processing, and using information [5]. A prevalent platform for seeking and sharing health information alongside breaking news stories is X (formerly known as Twitter) [6].

Specifically, sense-making and social media usage during the COVID-19 pandemic have received much attention from scholars in various fields, including sociology, psychology, communication, and information systems [4,7,8]. Studies in this area have investigated how individuals use social media to make sense of the rapidly changing and complex situation posed by the COVID-19 pandemic. Research has shown that social media can be essential in providing access to information, shaping public opinion, and influencing behavior during a crisis.

One strand of research has investigated how individuals use social media to gather information and make sense of the pandemic. Studies have shown that social media, such as X, can serve as an essential source of information for individuals, especially during a crisis when traditional sources of information may be limited or unreliable. Social media also enables individuals to seek out information from various sources, which can help them form a more nuanced understanding of the situation [9].

Another strand of research has investigated the role of social media in shaping public opinion and behavior during the pandemic. Studies have shown that social media can be an essential factor in shaping public opinion and influencing individuals' decisions about things like their behavior and whether to seek medical treatment or follow recommended guidelines [10,11]. Despite this body of research, there are still some crucial gaps in our understanding of the relationship between sense-making and social media usage during the COVID-19 pandemic. More research is needed to investigate the role of social media in shaping public opinion and behavior, particularly in diverse populations and geographic regions. Additionally, there is a need for more cross-country digital research on how individuals use social media to make sense of the pandemic, including how they navigate and interpret the vast amounts of information available on social media platforms. While studies have explored the impact of social media on public opinion and behavior, more research is needed to understand how X influences public opinion and behavior during uncertainty and crises such as COVID-19 [12].

This study seeks to analyze data on X related to the 3 phases of COVID-19 and the response of citizens across different countries on X. Our study focused on examining data from Italy, the United Kingdom, and Egypt. Italy has a national health system and is a member of the European Union. The United Kingdom is a country that also has a national health system and has recently left the European Union. Egypt does not have a national health system nor is it a member of any allied institutions. For these reasons, these 3 countries provide an interesting comparison point. Our study would uncover the differences in public perceptions and cultural attitudes toward crisis information on social media. The discourse from certain countries may also vary depending on culture, freedom of speech restrictions, and events.

In order to contextualize the developmental differences between Egypt, Italy, and the United Kingdom, we made reference to the Human Capital Index [13]. This is a quantitative measure of development based on health and education outcomes. According to this index, Italy scores 0.73, the United Kingdom 0.78, and Egypt 0.49. The index highlights how there is a substantial developmental gap between Italy and Egypt (0.73 vs 0.49) compared to the gap between Italy and the United Kingdom (0.73 vs 0.78). The significant difference between Egypt's Human Capital Index and that of Italy and the United Kingdom substantiates our classification of Egypt as a developing country relative to these 2 more developed nations. This cross-country variation allows us to explore how diverse sociocultural contexts and government responses influence citizen engagement with COVID-19 information on social media. Italy, the United Kingdom, and Egypt had varied timelines and policies. Italy responded with strict early lockdowns, coupled with economic support programs intended to mitigate financial strain. The United Kingdom initially adopted a more gradual approach, balancing public health measures with economic interests before implementing more extensive restrictions as the pandemic progressed. Egypt, on the other hand, maintained a comparatively limited governmental response, with religious institutions playing a crucial role in disseminating health guidance and encouraging precautionary behaviors.

### Research Aims

This study examines the variation of public attention and interaction with COVID-19 by analyzing data from X. It tries to estimate how COVID-19 has captured the attention of citizens across Italy, Egypt, and the United Kingdom. The theoretical lens of the study is sense-making. More specifically, the research questions (RQs) of this study are as follows (Textbox 1).

**Research aims of this study.**

- RQ1: To develop an understanding of public views shared on X toward COVID-19 across Egypt, Italy, and the United Kingdom.
- RQ2: To compare the response of citizens toward COVID-19 on X across Egypt, Italy, and the United Kingdom.
- RQ3: To examine the concept of sense-making concerning community responses to COVID-19 in Egypt, Italy, and the United Kingdom.

This study contributes to the existing body of knowledge by providing a cross-cultural analysis of public sense-making processes and reactions. This remains crucial for understanding how communities adapt to health crises. While the data were collected during the early stages of the pandemic, the lessons learned are still applicable to current and future public health challenges, particularly in designing culturally sensitive communication strategies and mitigating misinformation. Moreover, to the best of our knowledge, this is the first empirical investigation that systematically examines public discourse on X during the COVID-19 pandemic across Egypt, Italy, and the United Kingdom, offering a comparative perspective.

### Literature Review: COVID-19 and Social Media Research

A wide range of research on the COVID-19 pandemic, which draws upon X datasets, has been conducted. A recent study proposed several algorithms to estimate work engagement. Other studies have ranged from the identification of misinformation [14], examining public views and opinions related to various issues around the pandemic [15], and research related to epidemiology [16]. In one of the earliest studies examining misinformation on X, the author examined how a conspiracy theory linking COVID-19 to 5G spread on X [16]. Social network analysis was used alongside a content analysis to identify key users, content, and websites related to the conspiracy. Other research has also examined the relationship between fact-checking and misinformation [17]. The study found that fact-checks tend to appear after the spread of misinformation but that their ability to reduce the spread of misinformation is limited. Furthermore, they found that topics such as conspiracies are more resistant to fact-checkers.

Scholars have also analyzed X data to better understand perceptions of face masks [18]. In one study, the authors analyzed the content of the posts that talked about the vaccine and categorized them into these categories (provaccine, antivaccine, and neutral). Consequently, they found that the antivaccine posts use the sarcasm method, and some of them come from bots, while the pro-vaccine posts depend on documented information [19]. It is worth noting that over time, trust in the information spread on the internet is affected by the recipient's political affiliation [20].

Several studies have conducted research looking at COVID-19 in relation to other platforms. Although not a study on social media data itself, Wang et al [21], through a web-based survey posted on Facebook, found that Taiwanese citizens were mostly using the internet for their health information. This is an important finding as it highlights that web-based sources are likely to be the most viewed sources of information for a subset of the population. In another study, Sesagiri Raamkumar et al [22] examined the outreach efforts of public health authorities on Facebook in Singapore, the United States, and England. They found that the most common theme among all posts was those related to prevention and safety measures. It is also important to mention that outside of COVID-19 research, X has also been used across a range of disciplines in innovative ways. For example, X has been used to identify the political affinity of web-based entities through X followers and for the automatic identification of eyewitness messages on X at the time of natural disasters.

Although previous research has examined content from social media related to COVID-19, there is a lack of empirical work that has compared user views across different geographical locations using time as a moderating variable. Our proposed study hopes to address this research gap and contribute to the wider public health literature by developing an understanding of social media user views across 3 countries.

### Theoretical Background

We define the term sense-making as a process through which individuals aim to develop an understanding of events that may be confusing, uncertain, and violent by interacting with others [20-25]. In recent years, scholars have studied the concept of sense-making about interactions on social media platforms. For instance, a study by Zolyomi et al [5] examined the #ActuallyAusistic hashtag on X and found that autistic individuals used X to engage with sense-making to understand themselves better. In this context, sense-making was applied to construct identities in relation to individual social groups, employment, education, and the use of technology. Another study [22] focused on X collective sense-making that was examined during the Berlin terrorist attack. The study found that X users made sense of the events that were occurring on X. The study highlighted how collective behavior occurred during the aftermath of a terrorist attack. There are many definitions and discussions of social media within academic literature [26]. A popular definition notes that they are “a group of internet-based applications that build on the ideological and technological foundations of Web 2.0 and that allow the creation and exchange of user-generated content” [27].

Previous research has also examined sense-making on social media during other extreme events. For instance, Stieglitz et al [23] analyzed X data related to 3 events: the Sydney Lindt Café siege (2014), the Germanwings plane crash (2015), and the Brussels terror attacks (2016). They found that there were differences in how users communicated with one another across the 3 events. The authors note that citizens may use social media during extreme events to speak with one another and make sense of an event.

The researchers [24] noted that the information a person receives significantly impacts his behavior. Furthermore, they found a strong relationship between misinformation spread on social media and following preventive measures with the COVID-19 epidemic. Therefore, the researchers recommended publishing the correct news and confronting the misinformation to improve the citizens' behavior.

## Methods

### Study Design

**Figure 1:**
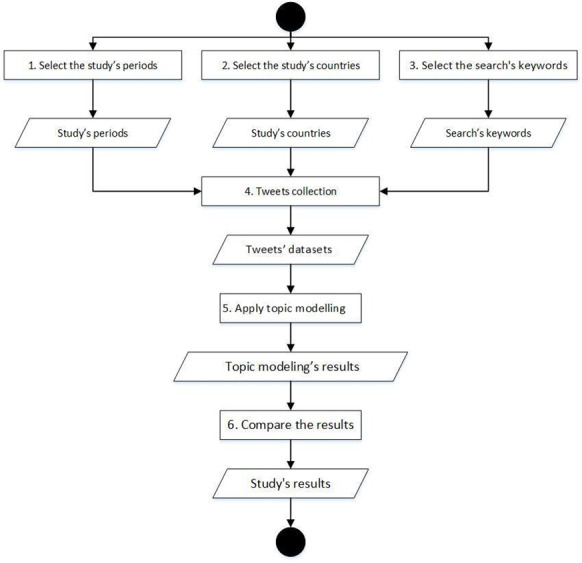
Overview of the 6 phases of our methodology.

The study was designed and executed in 6 phases, and a summary of these can be found in Figure 1, which provides a visual summary of our research design. Our study was pragmatic, inductive, and exploratory in nature, drawing upon the theoretical lenses of sense-making.

### Time Period Selection

COVID-19 appeared toward the beginning of 2020, and up to early 2021, there were 3 key periods for the pandemic, which can be characterized as follows: the emergence phase; during the initial lockdown; and when cases began to reappear during the second winter lockdown.

The difference between the 3 periods has been categorized based on the COVID-19 spreading and the political and governmental procedures and interventions that are undertaken. Therefore, the first period indicates the period of the virus' emergence and spreading, which took almost 2 months to be recognized as a pandemic by the World Health Organization. The second period includes the period of strict decisions that governments have made, which, in some countries, have reached total lockdown. The last period consists of the period of containing the virus and easing the severity of government restrictions. Since countries have responded to the pandemic situation differently, this study has tried to select countries that cover the spectrum of different situations.

All in all, we used the same time frames for Italy, the United Kingdom, and Egypt to analyze the influence of Italy’s early pandemic responses on global strategies. This uniform approach allows for comparative insights into how different regions adapted measures pioneered by Italy, facilitating an understanding of both reactive and locally influenced responses. In addition, the gap between the studied periods allows us to examine the adjustments and preparations made after initial lockdowns and before the resurgence of the virus. This helps in understanding the balance countries maintained between economic reopening and health safety, which is crucial for evaluating the effectiveness of pandemic management over time.

The overview of the time intervals across these phases is shown in Table 1, which shows our 3 time periods and their associated time intervals for which data was collected.

**Table 1:** Overview of the 3 time periods examined.

Periods	Time intervals
The virus' emergence	From February 15 to March 31, 2020
Lockdown	From April 1 to May 30, 2020
Virus spreading	From December 1, 2020, to January 15, 2021

### Keyword Strategy

A group of keywords most used by X users when conversing about COVID-19 was chosen using the website Getdaytrends. This website shows the most used hashtags during a specific time, including the number of posts attached to such hashtags for a particular country. This process allows for getting the most posts about COVID-19. Table 2 provides an overview of the keywords used to retrieve data for the respective countries examined.

**Table 2:** Overview of the keyword strategy to retrieve data.

Hashtag	Country
COVID_19	Italy, the United Kingdom, and Egypt
COVID-19	Italy, the United Kingdom, and Egypt
COVID19	Italy, the United Kingdom, and Egypt
COVID2019	Egypt
coronavirus	Italy, the United Kingdom, and Egypt
كوفيد19 (en^a^: COVID19)	Egypt
كوفيد_19 (en: COVID_19)	Egypt
كرونا (en: CORONA)	Egypt
كورونا (en: CORONA)	Egypt
فيروس_كورونا (en: CORONA_VIRUS)	Egypt
كورونا_مصر (en: CORONA_EGYPT)	Egypt

^a^en refers to the English language.

### Collecting X Data

Posts were collected in Python via the tweepy library. A requirement when collecting posts was to be written in the country's language and posted by its inhabitants. In order to fulfill these 2 conditions, some parameters were determined in the collection's algorithm. The algorithm uses the “lang” parameter to determine the post's language and the “geocode” parameter to determine the users' position when they have posted. Since the “geocode” parameter requires latitude and longitude, we have collected this information for all cities in our 3 countries via Google Maps. Consequently, the collection steps were executed as a loop for each keyword the algorithm executes for each city. The posts collected contain at least 1 hashtag of the search's keywords list.

The posts from Italy were filtered based on the language, assuming that the Italian language is unlikely to be used significantly outside of Italy (ie, to represent another county or region). However, filtering the posts based on location and language was used to collect the posts from Egypt and the United Kingdom, as English and Arabic could represent other regions.

Posts that were retrieved contain at least 1 keyword from Table 2. Table 3 shows the number of posts collected for each country across the 3 time periods examined. It is important to note that the number presented in Table 3 represents the final collected posts after applying 3 separate types of filters on the collected posts, which were as follows (Textbox 2).

**Table 3:** Overview of data retrieved across phases and countries from X.

Country	Final collected posts, n		
	The virus' emergence (phase 1)	Lockdown (phase 2)	Virus spreading (phase 3)
Italy	98,475	100,746	85,291
United Kingdom	84,675	97,842	78,461
Egypt	75,712	91,846	42,167

**Filters applied to collected data.**

- All repeated posts were deleted using the post ID.
- All posts that had the same text were deleted.
- All posts with less than 20 characters were deleted.

### Application of Topic Modeling

Topic modeling algorithms are frequently used to analyze posts. This method was selected in this study due to the volume of posts being analyzed. One of the most important types of these algorithms is latent Dirichlet allocation (LDA) [25]. The LDA model is a generative model used in natural language study, which allows for extracting topics from a set of source documents. Each document is considered a group of words that form one or more latent topics when combined [26]. A particular distribution of terms characterizes each topic. LDA's generative process is based on analyzing the text's data (text mining). Word combinations are considered as random variables.

The LDA algorithm was used to identify frequently used conversation topics in the corpus of data collected within the COVID-19 context. The selected number of topics (T) were extracted from a collective dataset (C). Probability (Pi) of inclusion of individual posts (Ci) from the dataset can be represented as:

Probability (Ci ∈ ti) = Pi

Collective posts C = {C1, C2, C3…,Cn}, where topic distribution T = {t1, t2, t3,…,tn}

Our topic models generated a topic matrix and the likelihood of individual topic strength. The bag of words constructed within the matrix can be designated as n-grams. In our case, a bigram model was selected due to its popularity in strengthening topic cohesion and topic relevance. We experimented with the optimum topic strength value up to the recommended 30. Several rounds of experimentation showed that the thematic importance of collective sense-making could be best captured between 2 and 7 sets of topics using bigram values.

The application of the topic modeling is divided into 3 steps. The first step is preparing the dataset to train the model, where each post is treated as a document. The second step is selecting the number of topics that are identified. The analysis will be conducted more than once, as each time, a different number will be determined for the number of topics that will range between 2 and 10. After each iteration, the coherence value will be extracted, indicating the model's efficiency [26]. Afterwards, the number of topics used in the model with the highest coherence value will be selected, and if all topics represent more than 5% of the dataset, we will decrease the topic numbers to achieve this condition. In the third step, the LDA model will be repeated, confirming the number of topics that are selected in the previous steps. Then, the LDA model will be used to extract a list of words representing each topic; consequently, the word lists will be used to describe each topic's connotation. Moreover, the LDA model classifies posts by their represented topic; consequently, the percentage each topic represents within the database will be calculated.

### Identification of Topics

After completing the previous step, the study will have developed results for 9 datasets, 3 for each country. The results for each country are discussed separately and will be compared later. We have obtained 9 independent topic models by applying the LDA model to each country's datasets. Each topic model returns the number of topics representing the dataset and the top words of each one. In the next section, we give a label and a description for each topic.

Finally, the study used LDA and bigrams to analyze large-scale social media posts, aiming to extract meaningful topics. LDA was chosen for its effectiveness in handling vast textual data, while bigrams enhanced the contextual relationships between words. Despite challenges like broad topics and tweet brevity, LDA proved suitable for capturing public discourse. The paper defines “sense-making” as how individuals interpret public health information, especially during crises. The topics derived through LDA reflect public interpretations of COVID-19, and manual labeling by experts ensured accurate insights into public responses.

### Ethical Considerations

This research was covered by ethical approval gained from Newcastle University (ref: 1067/2020). Moreover, our study is conducted on aggregate data, and we do not draw attention to any individual users or publish any identifiable information.

### Use of Tools to Support Proofreading

We drew upon large language models such as ChatGPT (version 4o) for basic proofreading as well as tools such as Grammarly to identify any errors in writing and sentence construction. However, no tools were used to generate text of their own.

## Results

Table 4 provides an overview of the results of the study.

**Table 4:** Overview of the key themes, subthemes, countries, and time periods.

Overall theme and topics or subthemes		Country	Period
**The general situation**			
	The general situation in Italy	Italy	The virus' emergence (phase 1)
	Region of Lombardy	Italy	Lockdown (phase 3)
	General situation	Italy	Virus spreading (phase 3)
	General situation	Egypt	Lockdown (phase 3)
	General situation	Egypt	Virus spreading (phase 3)
	The situation in Europe	United Kingdom	Virus' emergence (phase 1)
	General situation	United Kingdom	Lockdown (phase 3)
	General situation	United Kingdom	Virus spreading (phase 3)
**Protection**			
	Protection	Italy	The virus' emergence (phase 1)
	Quarantine process	Italy	Virus spreading (phase 3)
	Religious side	Egypt	Virus spreading (phase 3)
	Prepare for a pandemic	United Kingdom	Virus' emergence (phase 1)
	Lockdown	United Kingdom	Virus' emergence (phase 1)
**Media**			
	Media	Italy	The virus' emergence (phase 1)
**Virus**			
	Virus	Italy	Lockdown
	Virus mutated	Italy	Virus spreading (phase 3)
**Economic**			
	Economic status	Italy	Lockdown (phase 3)
	The European role	Italy	The virus' emergence (phase 1)
	Economic impact	United Kingdom	Lockdown (phase 2)
**Vaccine**			
	The vaccine	Italy	Lockdown (phase 2)
	The vaccine	Italy	Virus spreading (phase 3)
	The vaccine	Egypt	Virus spreading (phase 3)
**Religion**			
	Places of worship	Egypt	Virus' emergence (phase 1)
	Evoking historical events	Egypt	Virus' emergence (phase 1)
**Lack of confidence in the government**			
	Lack of confidence in the government	Egypt	Virus' emergence (phase 1)
	Demanding the dismissal of the Minister of Health	Egypt	Lockdown (phase 2)
**Medical staff**			
	Medical staff	Egypt	Lockdown (phase 2)

### Content on X in Italy (Phase 1)

This information in Table 4 can be used to give the following descriptions for content in Italy during phase 1:

- Topic 1: General situation in Italy:
  The virus began to spread increasingly in northern Italy, especially the Lombardy region, which caused an increase in the number of patients in need of intensive care and increased the number of deaths.
- Topic 2: European role:
  With the start of the lockdown, citizens sensed the extent of the economic damage that the virus might cause. They also sensed the Italian government's inability to deal with these damages on its own. For this reason, discussions noted how the European Union must intervene as soon as possible and help citizens and companies alike.
- Topic 3: Protection:
  The virus is unknown by specialists, who cannot predict the mechanism of its spread. Therefore, all preventive measures must be taken, especially wearing masks when leaving the house. Consequently, the government has been asked to secure many masks to meet citizens' needs and distribute them free, as did a region of Tuscany.
- Topic 4: Media:
  The media, in their various forms, played a significant role in reporting the stories and sacrifices of medical personnel in the face of this virus; besides, it focuses on the political disagreements between the government and the opposition.

### Content on X in Italy (Phase 2)

This information in Table 4 can be used to give the following descriptions for content in Italy for phase 2:

- Topic 1: Region of Lombardy:
  Despite quarantine, the number of injuries in the region is still on the rise. This rise has been accompanied by an increase in the number of deaths and increased pressure on the health sector, which has become weak and unable to bear more patients.
- Topic 2: Virus:
  Some voices appeared on social media that deny the existence of the virus or claim to reduce its risk. For instance, some noted that the virus can be eliminated by taking certain vitamins and that the lockdown measures were created to destroy countries' economies. The presence of such voices prompted many to respond to them and prove that such claims were incorrect.
- Topic 3: Economic status:
  With the increase in the quarantine period, fears of citizens' deteriorating economic situation and the fear of losing their jobs began to appear. A number of citizens noted that they did not have enough money to buy basic necessities.

### Content on X in Italy (Phase 3)

This information in Table 4 can be used to give the following descriptions for content in Italy for phase 3:

- Topic 1: Virus mutated:
  A new virus mutation in England was discussed, and users hypothesized that there would be an increased transmission. Meanwhile, the arrival of the new mutation to Europe increased fears, and the demand to close borders started.
- Topic 2: Quarantine process:
  The government enacted a new law to regulate the quarantine process, giving color to each region according to the data on the epidemic. Each color symbolizes different restrictions on the inhabitants of the areas. This law was difficult for citizens to understand, especially the differences in color restrictions.
- Topic 3: The vaccine:
  The government developed a nationwide plan to vaccinate citizens, which began with the arrival of the Pfizer vaccine. X users noted that the Lombardy region should be provided with more doses to cover faster. Concerns resurfaced after Pfizer announced a delay in reaching the agreed quantities.
- Topic 4: General situation:
  The daily reports of the number of infections were being actively monitored by citizens. X users also requested that schools remain open regardless of the number of casualties.

Sense-making during the COVID-19 crisis started with wider discussions without a clear focus on selective topics. The trend eventually became more fixated on the cause and evolution of the crisis (SARS-CoV-2) and prevention measures (quarantine and vaccine).

### Content on X in Egypt (Phase 1)

This information in Table 4 can be used to give the following descriptions for content in Egypt for phase 1:

- Topic 1: Places of worship:
  The first measure to prevent the virus was to close the places of worship, which sparked a great debate among citizens, especially among the Muslim community members, due to the approaching month of Ramadan. Some of the users demanded for mosques not to close, and some insisted on closing the mosques due to the overcrowding during the month of Ramadan.
- Topic 2: Lack of confidence in the government:
  The government was not transparent in handling the epidemic, which caused citizens to distrust the reality of the reports, especially the numbers of infections. Besides, many citizens accused the government of spreading false news about the virus.
- Topic 3: Evoking historical events:
  Many invitations have appeared for the obligation to adopt the quarantine even if the government did not impose it. Indeed, these invitations were based on epidemics that occurred during the Prophet Muhammad's time and how he ordered people not to go to places where the epidemic was spreading and not leave the place of the epidemic for those in it now of its spread.

### Content on X in Egypt (Phase 2)

The information in Table 4 can be used to give the following descriptions for content in Egypt for phase 2:

- Topic 1: Medical staff:
  With the increase in the number of cases, hospitals' and medical staff's pressure increased, and they began to work as robots day and night. Meanwhile, the media highlighted the medical staff's sacrifices and the deaths that occurred among them.
- Topic 2: General situation:
  Hospitals are no longer able to receive more injured patients. They experience difficulty from shortages in equipment, which has caused terror among citizens who demanded the government to impose a complete lockdown.
- Topic 3: Demanding the dismissal of the Minister of Health:
  From many Egyptians' point of view on X, the minister was not up to the responsibility, and because they did not hire more doctors and nurses. And some Egyptians went to accuse the minister of killing patients.

### Content on X in Egypt (Phase 3)

This information in Table 4 can be used to give the following descriptions for content in Egypt for phase 3:

- Topic 1: The vaccine:
  Jordan obtained the Chinese vaccine and started the vaccination campaign. Accordingly, there was a campaign beckoning for the government to follow Jordan's steps and to be able to limit the spread of the epidemic.
- Topic 2: General situation:
  The number of injuries increases daily, and the number of deaths increases. Also, problems increase in hospitals. One of the most serious of these problems was the lack of oxygen in Al Hasina Hospital, which caused the death of a group of patients in the intensive care unit.

As opposed to a developed nation like Italy, in Egypt, collective sense-making was narrowly focused during phase 1. In the absence of clear scientific logic, during the initial days of the crisis, people relied on religious and historical analogies. As time progressed, people started to understand the importance of preventative measures like vaccines, yet overall discussions were identified to become broad and dispersed as time progressed.

### Content on X in the United Kingdom (Phase 1)

This information in Table 4 can be used to give the following descriptions for content in the United Kingdom during phase 1:

- Topic 1: Prepare for a pandemic:
  The people are sure of the epidemic's arrival; therefore, they declared the importance of preparing for it and strengthening the health sector.
- Topic 2: The situation in Europe:
  Citizens followed the news of the epidemic in Europe with great apprehension. The virus moved quickly between the Union countries; consequently, they were sure that it would reach them.
- Topic 3: Lockdown:
  The government suggested not to close, which caused great fear among the citizens, who immediately demanded the government to apply the lockdown.

### Content on X in the United Kingdom (Phase 2)

The information in Table 4 can be used to give the following descriptions for content in the United Kingdom during phase 2:

- Topic 1: General situation:
  The number of injuries increased daily, and the number of deaths also increased. X users also note that problems have increased in hospitals.
- Topic 2: Economic impact:
  Citizens had a real state of terror of the economic effects of the epidemic. Accordingly, they turned to the government to ask it to adopt economic policies that help companies and individuals.

### Content on X in the United Kingdom (Phase 3)

This information in Table 4 can be used to give the following descriptions for content in the United Kingdom during phase 3:

- Topic 1: General situation:
  Daily reports are still the focus of citizens' attention, especially the news of virus mutation. In addition, the government's demands for economic aid continued.
- Topic 2: The vaccine:
  The vaccine's arrival marked a significant turning point for citizens, giving them a dose of hope that the virus could spread, which increased significantly.

Collective sense-making in the United Kingdom shows unique characteristics with the transition of time. During the initial days, public opinion was varied and dispersed in terms of preparation for the pandemic. Due to early interventions from the government and health authorities, many people were questioning the preparedness and viability of the public health care system and whether it could withstand the unexpected crisis. With the passage of time, discussions and opinions became more dispersed and polarized in terms of the economy (during phase 2). Finally, during phase 3, collective sense-making topics were largely focused on the vaccine and its scientific viability, along with other general issues in terms of economic and employability support.

### Comparing Responses

This section will compare the different phases and the resulting themes that emerged. There were differences and certain similarities in how X users made sense of the unfolding events. Table 4 provided an illustration of all the themes that emerged for the 3 cases within the periods of the study, and further insights can be extracted from it, as provided in Textbox 3.

**Insights from comparing themes.**

- It shows how citizens were following posting or reposting the pandemic news, including the number of new cases, deaths, fatality issues, etc.
- It shows how posts in Italy and the United Kingdom covered mainly prevention measures imposed by the governments in their countries, the extent of their validity, and the need to adhere to them. However, posts from Egypt mainly focused on religious arguments to encourage people to take protection measures to cover the void left by the government. This trend has been adopted when the people feel that their government does not implement adequate preventive measures. On the other hand, many posts demanded that the houses of worship not be closed so that the prevention measures could be easily applied according to their description.
- It can be argued that the media has played a crucial role in following up on spreading the virus and discussing preventive procedures and government involvement, especially in the United Kingdom.
- The posts in Italy and the United Kingdom called on their governments to provide economic aid or increase this aid to match the extent of the damage they experienced, while the Egyptians did not demand that might be justified by people's belief in the government's inability to follow the same European economic policy.
- The virus mutation aligned with a great concern for Italian citizens, especially the fear of returning to complete closure again.
- Egyptian posts showed their lack of confidence in the government in its ability to manage the crisis. Many posts claimed that the Minister of Health was unable to protect the doctors working in the first line of defense and questioned how it would be able to protect the people.
- Overall, the posts in the 3 countries found that the only way out of the pandemic would be to use vaccines that had been developed.

## Discussion

### Principal Findings

In response to RQ1, the results of our analysis have developed an understanding of the key views shared on X toward COVID-19 across Egypt, Italy, and the United Kingdom. Table 4 provides insight into the differing views that X users shared during this time. In response to RQ2, we found that there were differences and some similarities in how X users responded to X across Egypt, Italy, and the United Kingdom. We provided a comparison of the key discussions that took place across different countries and phases. In response to RQ3, it was found that X users used X to seek, generate, and process information collectively. Henceforth, collective sense-making on X was taking place, and it was found that there were differences in how people gave meaning to their experiences. During phase 1, X users conversed about topics and events that were occurring around them, which also continued within phase 2. During phase 3, as global news of the vaccine began to emerge, the collective discussions across all 3 regions appeared to be focused on news of the vaccine. This was significant news, affected all citizens in all countries, and was a potential way to navigate out of the pandemic.

This analysis of social media posts from the United Kingdom, Italy, and Egypt highlights distinct variations in how citizens across different cultural contexts responded to the COVID-19 pandemic. Our study found that this was driven partly by differing levels of governmental trust and state intervention. Previous work highlights that public trust in government (shaped by historical, cultural, and political factors) can dictate the perceived credibility of government-issued health advice [28]. For example, the prevalence of religious discourse in Egypt’s response aligns with studies that suggest that when formal institutional trust is low, people may turn to alternative social structures, such as religious communities, to interpret health risks and determine safe behaviors [29]. This has practical implications for policy makers who might need to engage non-governmental institutions. These could include religious leaders who could help to strengthen public adherence to health measures, and this could be particularly impactful in regions where government trust is low.

Furthermore, in Italy and the United Kingdom, the focus of social media posts on government-imposed prevention measures and economic aid highlights an expectation for government responsibility and support. Research has shown that public calls for government assistance in times of crisis often reflect a belief in the government’s role as a welfare provider [30]. This observation reflects policy implications in that governments with higher trust levels are likely to see more public demands for direct economic relief, as opposed to countries where citizens may not expect or request such support due to institutional limitations. For instance, economic support policies in Italy and the United Kingdom were closely scrutinized by citizens on social media, suggesting that clear communication on relief measures can help manage public expectations and reinforce trust. Meanwhile, posts in Egypt often revealed skepticism toward government actions, particularly regarding the health sector’s capacity to manage the pandemic. This finding highlights the need for targeted interventions to improve transparency and crisis management to build institutional trust, which is important for effective public health responses [31].

Regarding limitations, the study used the X Search API, which meant that certain posts might be missing from our analysis. Moreover, future research could seek to examine the influence of X and conduct an analysis of posts to identify users who were most influential by betweenness centrality across the 3 time periods. Further research could also seek to follow up with certain X users for in-depth qualitative interviews. Moreover, it is important to note that text-based social media data, such as posts from X, can reflect biases influenced by demographics, geographic location, and how users act on a daily basis. These factors may result in varying word choices and sentiment, even when discussing similar topics. For example, geographic differences can affect how happiness is expressed on the web [32], while daily routines and spatiotemporal context can shape sentiments related to specific locations or events [33,34]. It is important to acknowledge these biases because such factors could influence the patterns observed in our analysis. Additionally, the population of users who geo-tag their posts may not be representative of the larger population, which may introduce further limitations when generalizing results [35,36].

### Theoretical and Policy Contribution

**Figure 2:**
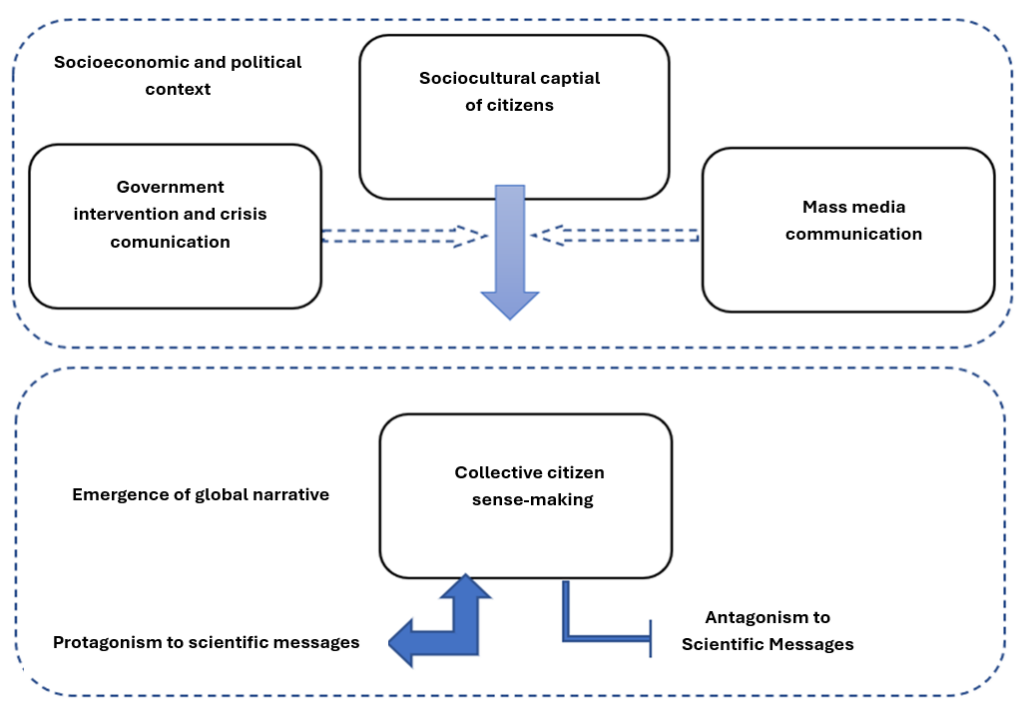
A visual overview of our proposed cycle of sense-making.

Our study found that users on X were observing and being presented with unfolding events that they would assess in relation to their current location and their own situation. These factors would then lead to sense-making among users, and they would repeat the cycle as they were being presented with new information. Figure 2 provides an overview of the process that was taking place.

In this study, we interpret “sense-making” as the process by which individuals can interpret and assign meaning to complex events such as the COVID-19 pandemic by engaging with public discourse and personal reflection. Unlike opinion-sharing, sense-making involves actively integrating new information to form coherent narratives [37]. While prior work, such as Stieglitz et al [38], explores sense-making during crises using sentiment and social network analysis, our study is different because it focuses on topic modeling to capture thematic patterns in discourse. Topic modeling, unlike sentiment analysis, enables us to explore the substantive content of conversations, identifying recurring themes beyond emotional valence and offering insights into how users contextualize the pandemic. Zolyomi and Snyder [39] applied a process model and manual coding to study sense-making in the #ActuallyAutistic community and focused on identity formation through qualitative analysis. However, our topic modeling approach is designed for large-scale analysis. This allowed us to reveal dominant narratives across diverse sociocultural contexts rather than process-tracing individual experiences. Social media platforms like X support collective sense-making during crises by providing spaces for users to negotiate meaning and validate perspectives [40]. By analyzing trends and patterns in sampled hashtags, we capture how individuals construct understanding beyond expressing opinions. This allowed us to reveal high-level themes that illuminate public sentiment across distinct institutional settings.

Based on our proposition derived from the findings of this study, “sense-making of events during COVID-19 on X differs based on the location of a user and their relationship with the location,” we have several practical insights, recommendations, and suggestions for public health authorities and governments (Textbox 4).

**Practical insights, recommendations, and suggestions for public health authorities and governments.**

- Users of social media platforms such as X are likely to have varying information needs at different points of a pandemic.
- Researchers analyzing X data may wish to segregate data based on location.
- The information needs of users are likely to differ based on their location.
- Users' sense-making and reaction process will take place in relation to their past experiences.
- Considering the above, information provided through the likes of X should be highly specific and targeted.
- Topic modeling methods can be used for social listening purposes to identify common queries and concerns for different locations.
- This information can feed into local and international health messaging.

Our research identifies unique patterns in how citizens across different regions use social media during pandemics, which has direct implications for targeted health communication strategies.

For instance, a public health official in Egypt, where our study noted significant religious and cultural influences on information dissemination, could leverage our insights to tailor messaging that resonates with local cultural contexts. During a pandemic, when misinformation can spread rapidly alongside the virus itself, understanding these nuances allows for the development of culturally sensitive, effective public health campaigns. Officials could use localized social listening tools, guided by our findings, to monitor real-time public sentiment and misinformation trends. This enables them to quickly address misconceptions and provide clear, trustworthy information specifically designed to engage and educate distinct audiences effectively.

Moreover, our study's suggestion to segregate data based on geographic and cultural lines could help these officials to identify and respond to emerging local issues more rapidly, potentially curbing the spread of the disease by improving compliance with health directives tailored to the community's prevalent beliefs and behaviors.

This actionable approach not only demonstrates the direct applicability of our findings but also reinforces the value of our research in enhancing public health responses through strategic communication tailored to diverse populations during critical times.

## Abbreviations

LDA: latent Dirichlet allocation
RQ: research question

## Data Availability

The datasets generated or analyzed during this study are not publicly available due to legal sharing restrictions in place by X.
